# Facile Synthesis of Triphenylamine Based Hyperbranched Polymer for Organic Field Effect Transistors

**DOI:** 10.3390/nano9121787

**Published:** 2019-12-16

**Authors:** Chinna Bathula, Alfred Bekoe Appiagyei, Hemraj Yadav, Ashok Kumar K., Sivalingam Ramesh, Nabeen K. Shrestha, Surendra Shinde, Hyun-Seok Kim, Heung Soo Kim, Lebaka Veeranjaneya Reddy, Arifullah Mohammed

**Affiliations:** 1 Division of Electronics and Electrical Engineering, Dongguk University-Seoul, Seoul 04620, Korea; chinnuchem@gmail.com (C.B.); hyunseokk@dongguk.edu (H.-S.K.); 2 Department of Energy and Materials Engineering, Dongguk University-Seoul, Seoul 04620, Korea; alfredappiagyei@gmail.com (A.B.A.); hemrajy@gmail.com (H.Y.); ashoksjc88@gmail.com (A.K.K.); 3 Department of Mechanical, Robotics and Energy Engineering, Dongguk University-Seoul, Seoul 04620, Korea; sivaramesh_74@yahoo.co.in (S.R.); heungsoo@dgu.edu (H.S.K.); 4 Division of Physics and Semiconductor Science, Dongguk University-Seoul, Seoul 04620, Korea; nabeenkshrestha@hotmail.com; 5 Department of Biological and Environmental Science, Dongguk University, Biomedical Campus, Ilsandong, Seoul 10326, Korea; shindesurendra9@gmail.com; 6 Department of Food Science and Technology, Yeungnam University, Gyeongsan 712-749, Korea; 7 Department of Microbiology, Yogi Vemana University, Kadapa (A.P.) 516003, India; 8 Institute of Food Security and Sustainable Science (IFSSA), Universiti Malaysia Kelantan Campus Jeli, Locked Bag 100, Jeli 17600, Kelantan, Malaysia

**Keywords:** benzo[1,2-b:4,5-b’]dithiophene, triphenylamine, Stille reaction, OFET

## Abstract

In this study, we reported the synthesis and characterization of a novel hyperbranched polymer (HBPs) *tris*[(4-phenyl)amino-*alt*-4,8-bis(5-(2-ethylhexyl)thiophen-2-yl)benzo[1,2-b;4,5-b’]dithiophene] (PTPABDT) composed of benzo[1,2-b:4,5-b’]dithiophene (BDT) and triphenyleamine (TPA) constituent subunits by A_3_ + B_2_ type Stille’s reaction. An estimated optical band gap of 1.69 eV with HOMO and LUMO levels of −5.29 eV and −3.60 eV, respectively, as well as a high thermal stability up to 398 °C were characterized for the synthesized polymer. PTPABDT fabricated as an encapsulated top gate/bottom contact (TGBC), organic field effect transistors (OFET) exhibited a p-type behavior with maximum field-effect mobility (µmax) and an on/off ratio of 1.22 × 10^−3^ cm^2^ V^−1^ s^−1^ and 7.47 × 10^2^, respectively.

## 1. Introduction

Solution processable π-conjugated semiconductors, a subset of organic materials, has progressively attracted much attention from both academic and industrial applications as the best candidate for lightweight, transparent, flexible electronic devices with less production cost for high throughput processing [1,2,3,4]. These materials have been fabricated as active organic layers in organic field-effect transistors (OFETs), organic photovoltaic (OPV) cells, and organic light emitting diodes (OLEDs) [5,6]. OFETs remain as the fundamental building block for flexible electronics [7,8]. Significant progress has enhanced device performance remarkably and currently considered as viable alternative to amorphous silicon-based transistors [9,10,11,12]. In spite of that, further fabrication methods and reliable device models are required for expansion of its application in sophisticated electronics [7,13]. This realization calls for comprehensive understanding into new materials design and synthetic concepts to control polymer energy levels [14,15], optimizing fabrication processes [16,17], and engineering devices so as to modulate semiconductor films and ensure effective alignment of the π-π bonds for charge delocalization and transport for high performance transistors [18,19].

Hyperbranched polymers (HBPs) represent highly branched three-dimensional (3D) macromolecules [20]. Their globular and dendritic architectures endow them with unique structures and properties such as abundant functional groups, intramolecular cavities, low viscosity, and high solubility [21]. HBPs can be facilely synthesized via a one-pot polymerization of traditional small molecular monomers or emerging macromonomers [22]. Benefiting from tailorable structures and correspondingly special properties, the achieved HBPs have been widely applied in various fields, including light emitting materials, nanoscience and technology, supramolecular chemistry, biomaterials, hybrid materials and composites, coatings, adhesives, and modifiers [23].

Benzo[1,2-b;4,5-b’]dithiophene (BDT) in particular is recognized for its symmetrical and planar structure which guarantees a more ordered π-π interactions to facilitate electron/holes transport. Moreover, BDT solubility properties and frontier energy levels are absolutely tunable since the central benzene core offers two sites for substituents attachments via alkylation reactions [24]. On the other hand, triphenylamine (TPA) is notably a strong electron donating unit as it possesses lone pair electrons on the central nitrogen atom bonded to three phenyl groups. This inherent characteristic makes it more applicable as a hole transport layer in OLEDs and photodiodes [25]. In contrast to BDT, steric hindrance effects cause TPA molecules to assume non-planar structure which suppress molecular agglomeration to a certain degree [26]. Triphenyl amine based copolymers have been explored as a nonvolatile transistor memories [27] and 2D covalent organic framework semiconductor thin films [28]. Hyperbranched TPA homopolymers and fluorene (fluorene trimer) cored TPA copolymers are prepared by one-pot Suzuki polycondensation [29]. We envisage that coupling TPA as a central unit with BDT moieties attached to the phenyl groups in a hyperbranched structure would allow for charge carrier transport for OFET application.

In this research, we present a communication on the synthesis and characterization of a hyperbranched conjugated polymer *tris*[(4-phenyl)amino-*alt*-4,8-bis(5-(2-ethylhexyl)thiophen-2-yl)benzo[1,2-b;4,5-b’]dithiophene] (PTPABDT) which features a benzo[1,2-b:4,5-b’]dithiophene (BDT) and star-shaped triphenylamine (TPA) by A_3_ + B_2_ approach, as a potential organic active layer for OFETs. As a proof of concept demonstration, the polymer was fabricated as an active layer in staggered top gate/bottom contact (TGBC) OFET. The device with PMMA as gate dielectric achieved hole mobility (µ_Fet_) of 1.22 × 10^−3^ cm^2^ V^−1^ s^−1^ and threshold voltage of −24.18 V at optimum annealing temperature of 100 °C. Taking into account of the fact that hyper branched polymer-based OFETs are uncommon, this study demonstrated that coupling triphenylamine and benzo[1,2-b:4,5-b’]dithiophene are promising moieties to build HBPs for organic electronic application.

## 2. Experimental Details

### 2.1. Materials

Triphenylamine, toluene (anhydrous 99.9%), dimethyl formamide (anhydrous 99.9%), tetrakis(triphenylphosphine)palladium (99.9%), methanol, acetone, hexane, and chloroform were received from Sigma Aldrich (Seoul, Republic of Korea). All chemicals were used as received unless stated otherwise. The monomer 2,6-bis(trimethyltin)-4,8-bis(5-(2-ethylhexyl)thiophen-2-yl)benzo[1,2-b;4,5-b’]dithiophene (BDT)[15] was prepared by previously described methods.

### 2.2. General Characterization

Nuclear magnetic resonance (NMR) spectra were recorded using Bruker DPX-300 NMR spectrometer (Bruker, Mundelein, IL, USA) at room temperature with TMS as internal standard. Chemical shifts of NMR were obtained in ppm (part per million) relative to the residual solvent peak for ^1^H NMR spectroscopy. The optical spectra by UV-absorption spectroscopy was run using T60 Visible spectrophotometer (Oasis Scientific Inc, Teylors, SC, USA) for both solution and film state. The number and weight average molecular weights of the polymers were determined by Gel Permeation Chromatography (GPC; Viscotek, Malvern Panalytical Ltd, Malvern, WR, UK) equipped with a TDA 302 detector and a PL-gel (Varian) column, using chloroform as the eluent and polystyrene as the standard. Thermogravimetric analysis (TGA) was performed under a nitrogen atmosphere at a heating rate of 10 °C/min with a Dupont 9900 analyzer (Delaware, Newark, DE, USA). Electrochemical properties were performed by cyclic voltammetry using in a three-electrode system with Ag/Ag+ electrode, Pt wire and 1.6 mm diameter Pt electrode as the pseudo reference, counter, and working electrodes, respectively. X-ray diffraction analysis (Agilent Technologies, Santa clara, CA, USA) was performed to identify the polymer phase transformations. 

### 2.3. Synthesis

#### 2.3.1. Synthesis of Tris(4-bromophenyl)amine

Triphenylamine (2.5 g, 10 mmol) was dissolved in 30 mL of chloroform after that the solution was cooled down to 0 °C and kept under constant stirring. Then 3 mL, 50 mmol of bromine, and Br_2_ was added dropwise in the dark. Once the addition was completed, the solution was stirred for 2 h for the reaction to be completed. Organic layer was washed with water and dried over the anhydrous sodium sulfate. The solvent was removed under reduced pressure. Crude product was recrystallized in CHCl_3_ -hot ethanol to obtain 1.67 g product, with 84% yield. ^1^H NMR (CDCl_3_, 300 MHz): δ (ppm) = 7.33 (d, 6H), 6.91 (d, 6H); ^13^C NMR (CDCl3, 300 MHz): δ (ppm) = 146.02, 132.50, 125.60, 116.05. Elemental analysis calculated (%) for C_18_H_12_Br_3_ N: C 44.85, H 2.51, N 2.91; found: C 44.81, H 2.47, N 2.93.

#### 2.3.2. Synthesis of Polymer Tris[(4-phenyl)amino-alt-4,8-bis(5-(2-ethylhexyl)thiophen-2-yl)benzo[1,2-b;4,5-b’]dithiophene] (PTPABDT)

Tris(4-bromophenyl)amine (62 mg, 0.12 mmol), bis(trimethyltin)-4,8-bis(5-(2-ethylhexyl)thiophe n-2-yl)benzo[1,2-b;4,5-b’]dithiophene(BDT) (174 mg, 0.19 mmol), and Pd(PPh_3_)_4_ (8 mg, 0.05 eq.) were added to a 50 mL round-bottom flask. Then, anhydrous DMF (2 mL) and anhydrous toluene (8 mL) were added. The polymerization was carried out at 110 °C under argon protection. After 48 h, the reaction mixture was cooled to about 50 °C and added slowly to a vigorously stirred methanol (50 mL). The polymer fibers were collected by filtration. Then the crude polymer was redissolved in chlorobenzene and precipitated again into methanol. The precipitate was then subjected to Soxhlet extraction with methanol, acetone, hexane, and chloroform. The final polymers were obtained by the evaporation of chloroform and precipitating in methanol. The polymer was filtered and dried in vacuum at 40 °C for 12 h. Red material is obtained in the yield of 76%. Mn = 11.8 K_Da_, Mw = 22.7 K_Da_, PDI = 1.92. ^1^H NMR (Figure S1) (CDCl_3_, 300 MHz): δ (ppm) = 7.52 (br, 2H), 7.30 (br, 2H), 7.24 (br, 2H), 6.88 (br, 2H), 6.50 (br, 2H), 2.75 (br, 4H), 1.54 (br, 2H), 1.28–1.1 (br, 16 H), 0.80 (br, 12H), Anal. calcd: C, 73.10; H, 6.84; N, 0.70; S, 19.35%; found C, 73.08; H, 6.80; N, 0.69; S, 19.31%.

### 2.4. Organic Field Effect Transistors (OFET) Fabrication

After conventional photolithography process of developing photoresist layer for source/drain contact electrode pattern on a glass substrate, a 3 nm thick Ni, and 13 nm thick Au metals were deposited thorough thermal evaporation to yield channel width to lengths ratio of 1:10. Sequentially, the glass substrates were cleaned in an ultrasonic bath with deionized water, acetone, and isopropanol for 10 min each. The glass substrates were treated with UV for 30 min. 5 mg/mL solution of PTPABDT polymer was spin-coated at 2000 rpm for 60 s. Then, they were thermally annealed at 100 °C, 150 °C, and 200 °C for 30 min each on a hot plate in a N_2_-purged glove box and then cooled to room temperature. 80 mg/mL solution of PMMA in n-butyl acetate (nBA) was spin-coated on the PTPABDT layer followed by baking at 80 °C for 2 h in the same N_2_-purged globe box to remove all residual solvents. OFET devices were completed by depositing 50 nm Al top gate electrode via thermal evaporation using a metal shadow mask. Electrical characteristics of the PTPABDT devices were measured using a Keithley 4200 parameter analyzer on a probe station.

## 3. Results and Discussion

### 3.1. PTPABDT Synthesis and Characterization

**Scheme 1:**
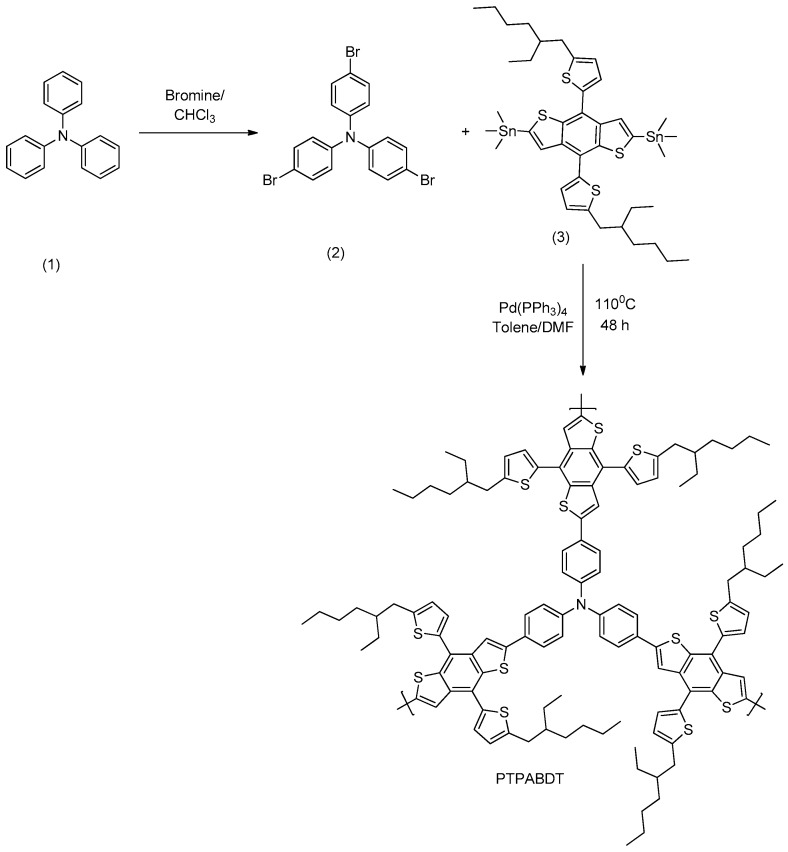
Synthesis of hyperbranched polymer.

**Figure 1:**
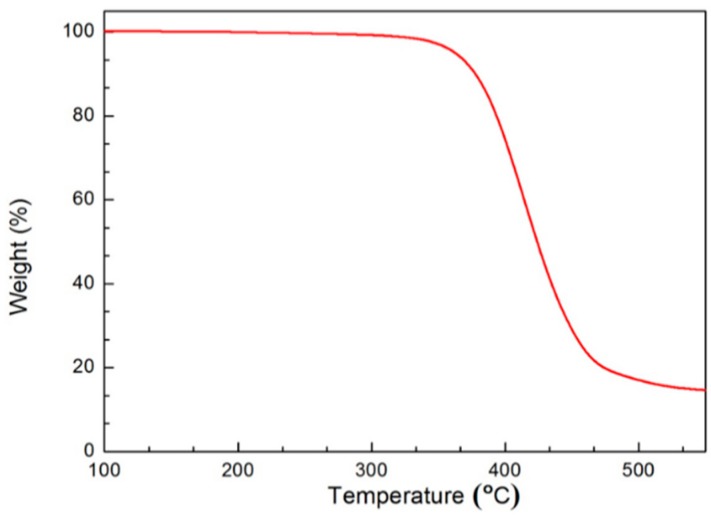
TGA plots for the polymer, obtained with a heating rate of 10 °C min^−1^ under an inert atmosphere.

Hyperbranched conjugated polymer PTPABDT which features a bifunctional benzo[1,2-b:4,5-b’]dithiophene (BDT) and star shaped trifunctional triphenylamine (TPA) were coupled together through Stille reaction as shown in Scheme 1. Polymer possessed good thermal stability and high solubility in common organic solvents such as chloroform, chlorobenzene, and o-dichlorobenzene. The number average molecular weight (Mn) was determined to be 11 kDa with a polydispersity index (PDI) (Mw/Mn) of 1.92 (Table S1), measured in chloroform solution (Figure S2). The polymer structure was confirmed by elemental analysis and ^1^H-NMR spectroscopy. In addition, we examined the thermal stability using the TGA analysis. The curve in Figure 1 showed a 5% weight loss temperature of 385 °C, indicating the possible exploration of the material in robust organic electronic application.

### 3.2. Optical Properties

**Figure 2:**
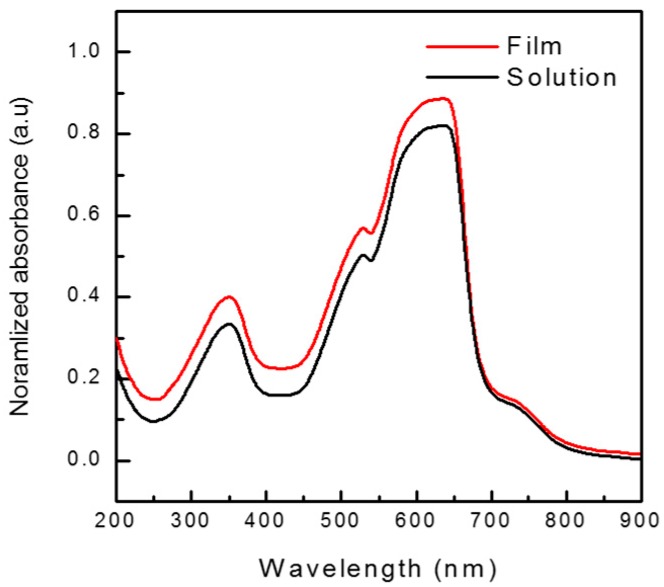
UV-vis absorption spectra of PTPABDT polymer both in solution (5 mg/mL) and in solid film state.

The spectra in Figure 2 depicts the optical characteristics of the PTPABDT polymer both in CB solution and on thin film. The absorption spectra both in solution and on thin film spans within a wavelength range from 200 nm to 900 nm with four distinct bands peaked around 350 nm, 530 nm, 640 nm, and 730 nm wavelength regions. These bands correspond to the localized π-π* stacking between the molecular orbitals and the intermolecular charge transfer between the TPA-BDT complex. In the solid state, the optical spectra became slightly broadened and showed a weak bathochromic shift revealed in the difference between the thin film and solution highest absorption peaks. The inconsiderable red-shift by 5 nm can be attributed to the interchain interaction and polymer aggregation in the solid states, though the shift is weak, yet it is desirable for enhanced charge transport. The optical band gap (E_g_^opt^) of PTPABDT was determined as 1.69 eV from the onset of the absorption edge (734 nm) of the film spectra by the relation E_g_^opt^ = 1240/(wavelength of the film absorption edge).

### 3.3. Electrochemical Properties

**Figure 3:**
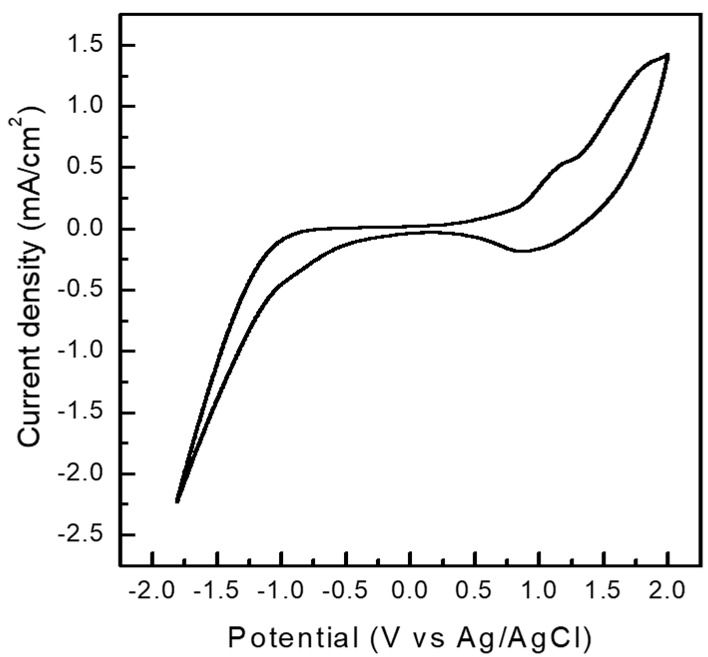
Cyclic voltammograms of PTPABDT in 0.1 M Bu_4_NPF_6_/CH_3_CN, scan rate 50 mV s^−1^, Pt working electrode.

Represented in Figure 3 is the cyclic voltammograms with oxidative (upshift) and reductive (downshift) redox reactions evident in the ends of the profile. The HOMO level of PTPABDT was deduced from the onset oxidation potential $\left(E_{\mathrm{ox}}^{\mathrm{onset}}\right)$ according to the following equation:

$$E_{\mathrm{HOMO}}=\;-\;\left(E_{\mathrm{OX}}-{\;E}_{\mathrm{FC}}+4.8\right)\left(\mathrm{eV}\right)$$

where E_FC_ is the potential of the reference redox, the Fc/Fc^+^ estimated as 0.25 eV vs. Ag/Ag+ in the same experimental condition. The HOMO level was determined to be −5.29 eV with LUMO level calculated as −3.60 eV from E_g_^opt^ = E_LUMO_ − E_HOMO_. The summary of the polymer material’s thermal, optical, and electrochemical properties are tabulated as shown in Table 1.

### 3.4. I-V Characteristics of PTPABDT Based OFETs Device.

**Figure 4:**
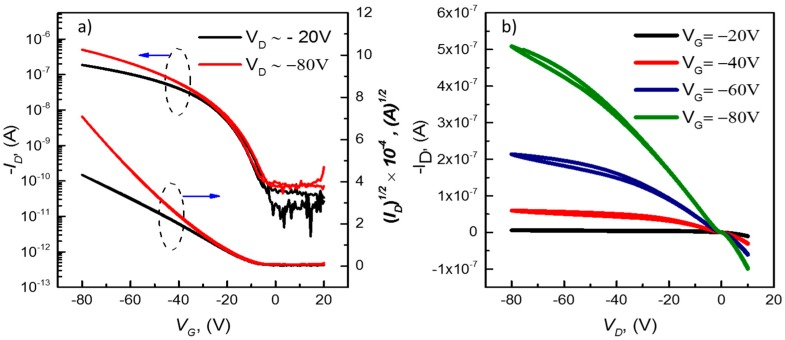
Representation of (**a**) transfer curve (**b**) output curve of PTPABDT based OFETs at the optimum annealing temperature.

The synthesized PTPABDT polymer was fabricated as an active layer in staggered top gate/bottom contact (TGBC) OFET to assess the charge transport properties of the polymer. Figure 4a represents the transfer curve obtained in the linear (V_D_ = −20 V) and saturation (V_D_ = −80 V) regimes. The corresponding output curve measured at V_G_ = −20 V to −80 V with −20 V increments illustrated in Figure 4b is a confirmation that the PTPABDT film device shows a strictly p-type semiconductor behavior. The electrical properties of PTPABDT can be ascribed to the high planarity of its π-π conjugation which reduces the charge trap density. Thermal annealing was employed as a technique to optimize device performance [30]. Shown in Figure S3, the highest drain current (I_D_) was recorded at 100 °C. The drain current level was improved by an order of magnitude by thermal annealing, which proved to be an effective means of enhancing device performance. We attribute orderly polymer crystal rearrangement and realignment to the enhance transistor performance. We employed X-ray diffraction to investigate polymer crystallinity for the as-cast and film at the optimum annealing temperature, 100 °C. Analysis showed no distinct crystalline peak in both films as shown in Figure S4. This directly infers an amorphous microstructure is observed in this polymer. The average field effect mobility and threshold voltage increased from (1.24 ± 0.592) × 10^−4^ cm^2^ V^−1^ s^−1^, −16.03 ± 2.05 V in pristine OFETs to (1.05 ± 0.125) × 10^−3^ cm^2^ V^−1^ s^−1^, −24.18 ± 0.50 V in OFETs annealed at 100 °C, respectively.

**Figure 5:**
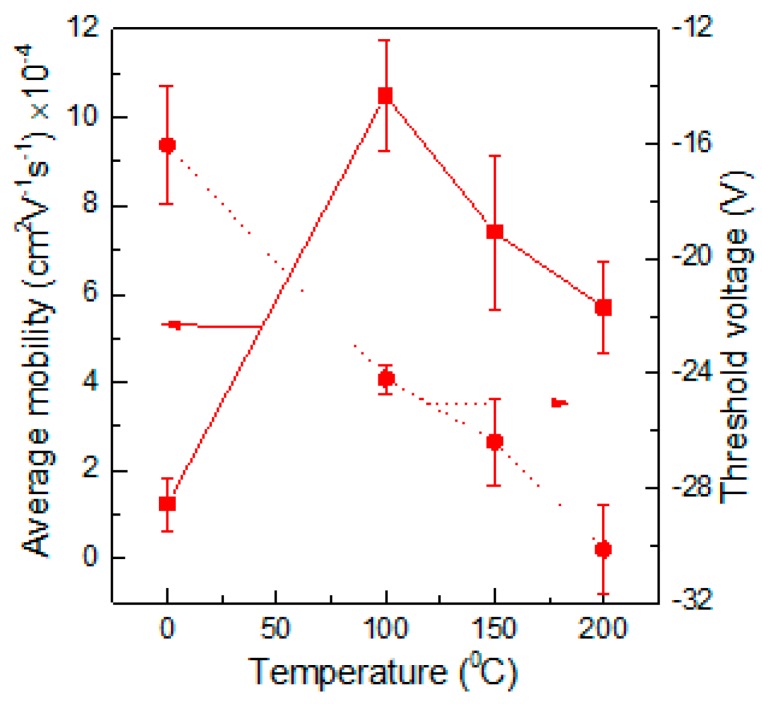
Graphical representation of the trend of average field-effect mobility and threshold voltage dependence on annealing temperature of the PTPABDT film.

Performance measurements of PTPABDT OFETs devices are summarized in Table 2. As illustrated in Figure 5, mobility gets increased from 0 to 100 °C and reduced gradually from 100 to 200 °C. Henceforth, we deduce that the optimum annealing temperature of PTPABDT film to achieve maximum performance to be 100 °C. The maximum hole mobility obtained are 2.08 × 10^−4^ cm^2^ V^−1^ s^−1^, 1.22 × 10^−3^ cm^2^ V^−1^ s^−1^, 1.02 × 10^−3^ cm^2^ V^−1^ s^−1^, and 7.28 × 10^−4^ cm^2^ V^−1^ s^−1^ at 0 °C, 100 °C, 150 °C, and 200 °C, respectively. The experimental mobility data is also comparable with the mobility of the triphenyl polymers reported in the literature [27,28,31] and are tabulated in Table 3.

### 3.5. Interfacial Electron Transport Properties: EIS-Nyquist Plot

**Figure 6:**
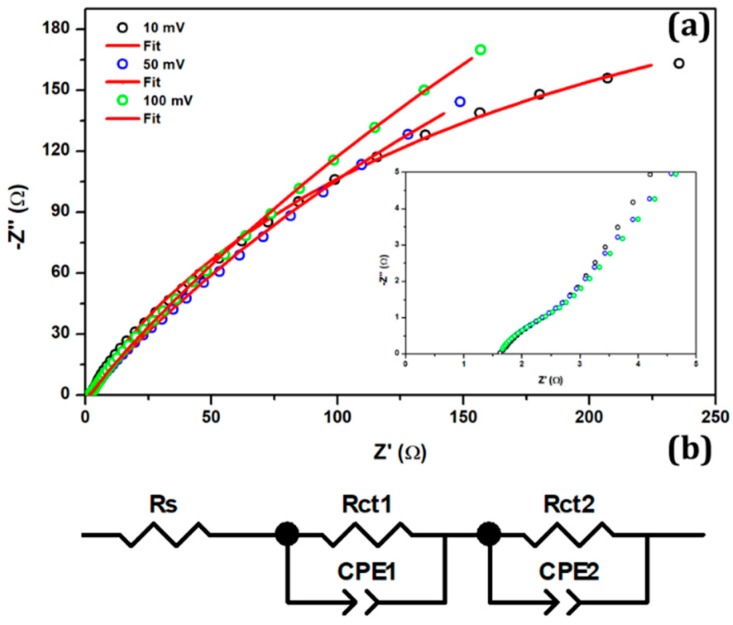
Electrochemical impedance spectroscopy (EIS) (**a**) Nyquist plots of triphenylamine based hyperbranched polymer with various applied bias potential and (**b**) Equivalent circuit model used for fitting.

The electrical properties of prepared triphenylamine based HBPs was further evaluated from the interfacial charge transport kinetics using EIS-Nyquist plot shown in Figure 6a. Analysis was done using two-electrode system for an applied bias potential of 10 mV, 50 mV, and 100 mV utilizing triphenylamine-based HBPs coated electrode as working and platinum plate as counter electrode. Here, 3M KOH solution was employed as supporting electrolyte. It reveals two semicircle arcs in the high and mid frequency region, where the real part of impedance is plotted against the imaginary part. An equivalent circuit model in Figure 6b was used to fit the obtained data and the electrochemical parameters were summarized in Table 4. R_s_ is the sheet resistance of electrodes utilized which are found to be c.a. 1.5 Ω. High frequency semicircle contributes charge transport properties at platinum electrode/KOH interface (R_ct1_), whereas the mid frequency semicircle is attributed to charge transport resistance (R_ct2_) at the grain interior of HBPs through its structure. Initially, for low applied potential of 10 mV, it shows very low charge transport resistance of 446 Ω at grain interior of HBPs which confirms the high electron mobility of the prepared sample. While increasing the applied potential to 50 and 100 mV, the charge transport resistance gets increased to 1003 Ω and 1361 Ω, respectively, owing to high planarity of its π-π conjugation in HBPs structure which reduces the charge trap density and electron mobility for higher bias potential.

## 4. Conclusions

In conclusion, the study presented provides understanding in the synthesis, characterization, and OFET application of a newly synthesized conjugated hyper branched polymer (HBPs) containing benzo[1,2-b:4,5-b’]dithiophene (BDT) and triphenylamine (TPA) moieties by A_3_+B_2_ protocol. PTPABDT polymer was analyzed for its inherent optophysical and frontier energy levels based on UV-spectroscopy and electrochemical measurements. The techniques showed polymer bandgap, HOMO, and LUMO levels estimation as 1.69 eV, −5.29 eV and −3.60 eV, respectively. Polymer possessed good thermal stability and high solubility in common organic solvents. The highest performance with hole mobility and on/off ratio ~1.22 × 10^−3^ cm^2^ V^−1^ s^−1^ and 7.47 × 10^2^ correspondingly at the optimum annealing temperature, 100 °C for 30 min was observed when the material was utilized as an active layer in OFETs. The observed photophysical and optoelectronic characteristics of the synthesized polymer suggest the hyper branched moiety is a promising candidate for the application in organic electronics. There are currently ongoing studies on this polymer design structures to further explore them in organic electronics application.

## Figures and Tables

**Table 1:** Summary of the thermal, optical, and electrochemical properties.

Polymer	Mn(K_Da_)/PDI ^a^	T*_d_* (°C) ^b^	λ_max_, Sol; Edge (nm) ^c^	λ_max_, Film; Edge (nm) ^c^	E_HOMO_ (eV) ^d^	E_LUMO_ (eV) ^e^	E_g_^opt^ (eV) ^f^
PTPABDT	11.7/1.92	398	635, 730	640, 734	−5.29	−3.60	1.69

^a^ Number-average molecular weight (Mn) and PDI of the polymers were determined by gel permeation chromatography (GPC) against polystyrene standards in chloroform as eluent. ^b^ Temperature resulting in 5% weight loss based on initial weight. ^c^ The UV-Vis absorption spectra of the polymers were measured in chloroform solution and thin film. ^d^ HOMO levels were determined from onset voltage of the first oxidation potential with reference to ferrocene at −4.8 eV. ^e^ LUMO levels were estimated from optical band gaps and HOMO energy levels. ^f^ Optical band gap was calculated from the UV-Vis absorption onset in film.

**Table 2:** Performance criteria of PTPABDT OFETs with different annealing temperatures.

Polymer	T (°C)	µ_max_ (cm^2^V^−1^s^−1^)	µ_avg_ (cm^2^V^−1^s^−1^)	V_T_ (V)	I_on/off_	SS (Vdec^−1^)
TPABDT	Pristine	2.08 × 10^−4^	(1.24 ± 0.592) × 10^−4^	−16.03 ± 2.05	7.62 × 10^0^	−8.92 ± 1.17
	100	1.22 × 10^−3^	(1.05 ± 0.125) × 10^−3^	−24.18 ± 0.50	7.47 × 10^2^	−13.41 ± 0.92
	150	1.02 × 10^−3^	(7.41 ± 1.75) × 10^−4^	−26.37 ± 1.53	4.67 ×10^2^	−15.22 ± 0.49
	200	7.28 × 10^−4^	(5.70 × 1.04) × 10^−4^	−30.13 ± 1.51	4.35 × 10^2^	−16.29 ± 0.77

The FET performance for 5–8 devices calculated in the saturation region from I_DS_ = (WC_i_/2L)µ(V_G_-V_T_)^2^ here I_DS_ is drain current, C_i_ is dielectric capacitance, V_G_ is gate voltage, V_T_ is threshold voltage, W and L are respectively channel width and length for (W/L = 1.0 mm/10 µm), with PMMA gate dielectric thickness and capacitance measured ≈500 nm and ≈6.2 nF cm^−2^, respectively.

**Table 3:** Mobility Comparison data.

Polymer	µ_max_ (cm^2^V^−1^s^−1^)	I_on/off_	Reference
PI(DAC-6FDA)	8.54 × 10^−2^	2.2 × 10^5^	[27]
PI(TPA-6FDA)	1.48 × 10^−3^	5 × 10^2^	[27]
Poly TB	3.0 × 10^−6^	850	[28]
PTTA2	4.86 × 10^−4^	1.24 × 10^2^	[31]
PTPABDT	1.22 × 10^−3^	7.47 × 10^2^	This work

**Table 4:** Interfacial charge transport resistance of hyperbranched polymer.

Bias Potential (mV)	R_s_ (Ω)	R_ct1_ (Ω)	R_ct2_ (Ω)	χ^2^ (× 10^−4^)
10	1.57	9.22	446	7.43
50	1.49	9.69	1003	15.9
100	1.48	9.51	1361	11.8

## Supplementary Materials

The following are available online at https://www.mdpi.com/2079-4991/9/12/1787/s1, Figure S1: ^1^H NMR of the polymer. Figure S2: GPC analysis of the polymer; Figure S3: Transfer output characteristics of PTPABDT based OFETs with different annealing temperatures during device fabrication; Figure S4: X-ray diffraction pattern at various annealing temperatures; Table S1: GPC analysis data of the polymer.
