# Supplementary material for: Facile Synthesis of Triphenylamine Based Hyperbranched Polymer for Organic Field Effect Transistors
Source: Nanomaterials (Basel). 2019 Dec 16;9(12):1787. doi: 10.3390/nano9121787 (PMC6955725; doi:10.3390/nano9121787)
Supplement: Supplementary file 1 [file nanomaterials-09-01787-s001.pdf]

Supplementary Materials

Facile Synthesis of Triphenylamine Based Hyperbranched Polymer for Organic Field Effect Transistors

Chinna Bathula [1], Alfred Bekoe Appiagyei [2], Hemraj Yadav [2], Ashok Kumar K [2], Sivalingam Ramesh [3], Nabeen K Shrestha [4], Surendra Shinde [5], Hyun-Seok Kim [1], Heung Soo Kim [3], Veeranjaneya Reddy [6,7,*] and Mohammed Arifullah [8,*]

[1] Division of Electronics and Electrical Engineering, Dongguk University-Seoul, Seoul 04620, Republic of Korea; chinnuchem@gmail.com (C.B.); hyunseokk@dongguk.edu (H.-S.K.)
[2] Department of Energy and Materials Engineering, Dongguk University-Seoul, Seoul 04620, Republic of Korea; alfredappiagyei@gmail.com (A.B.A.); hemrajy@gmail.com (H.Y.); ashoksjc88@gmail.com (A.K.K.)
[3] Department of Mechanical, Robotics and Energy Engineering, Dongguk University-Seoul, Seoul 04620, Korea; sivaramesh_74@yahoo.co.in (S.R.); heungsoo@dgu.edu (H.S.K.)
[4] Division of Physics and Semiconductor Science, Dongguk University-Seoul, Seoul 04620, Republic of Korea; nabeenkshrestha@hotmail.com
[5] Department of Biological and Environmental Science, Dongguk University, Biomedical Campus, Ilsandong, Republic of Seoul 10326, Korea; shindesurendra9@gmail.com
[6] Department of Food Science and Technology, Yeungnam University, Gyeongsan 712-749, Republic of Korea
[7] Department of Microbiology, Yogi Vemana University, Kadapa (A.P.) 516003, India
[8] Faculty of Agro Based Industry, Institute of Food Security and sustainable Agriculture(IFSSA), Universiti Malaysia Kelantan Campus, Jeli 17600, Malaysia
* Correspondence: lvereddy@yahoo.com (L.V.R.); aurifullah@umk.edu.my (M.A.)

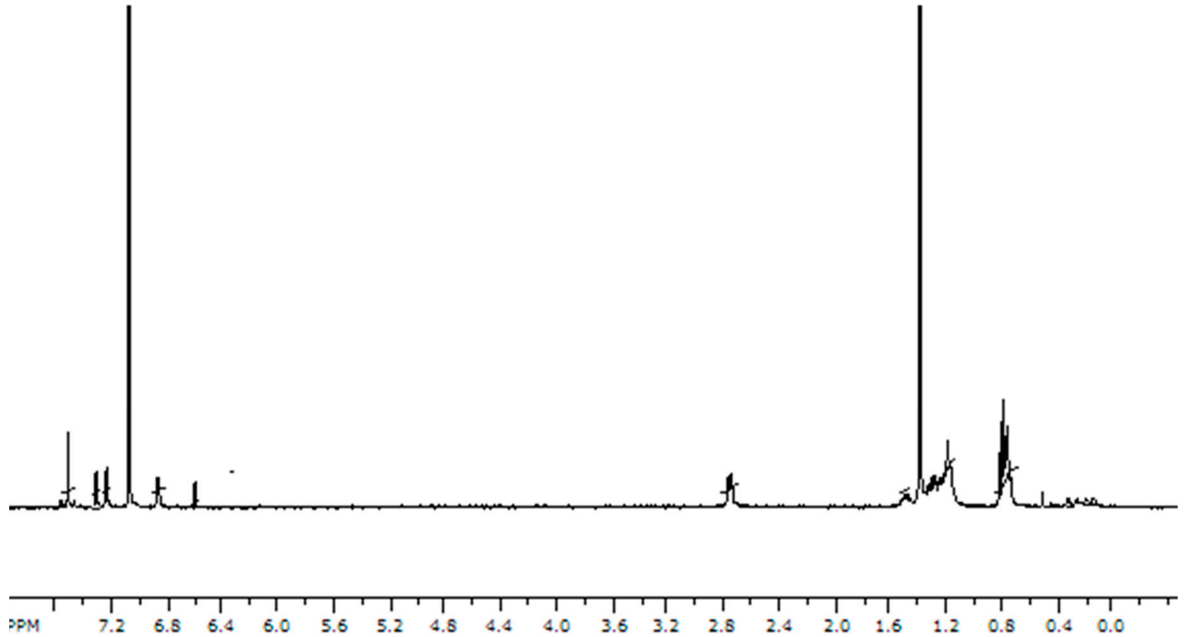

Figure S1. ^{1}H NMR of the polymer.

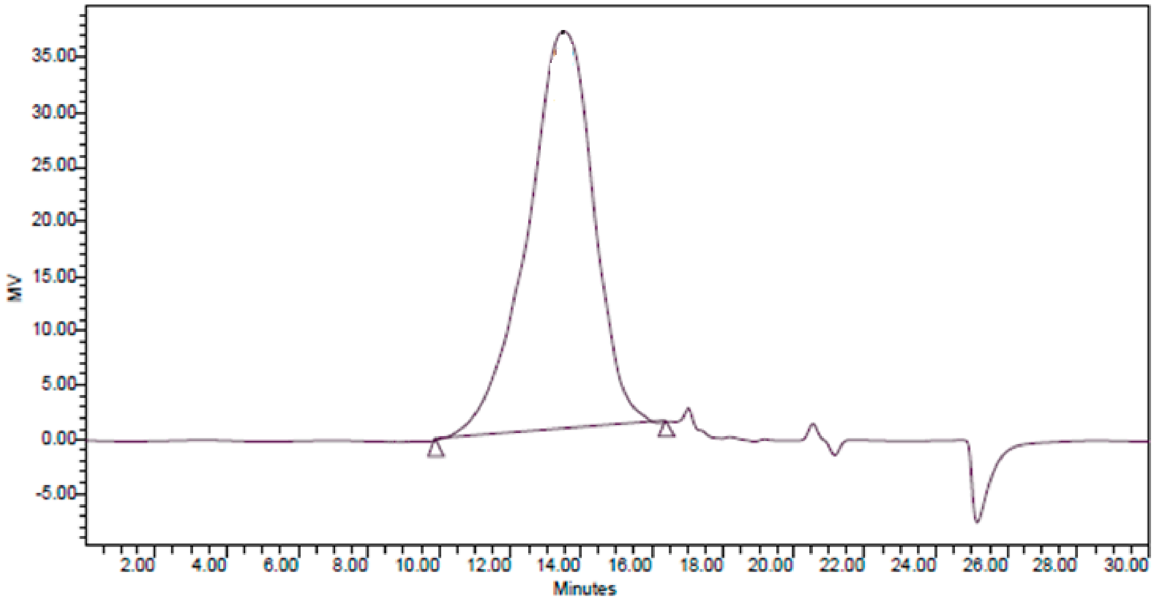

Figure S2. GPC analysis of the polymer.

Table S1. GPC analysis data of the polymer.

Polymer	Mn	Mw	PDI
PTPABDT	11805	22732	1.92

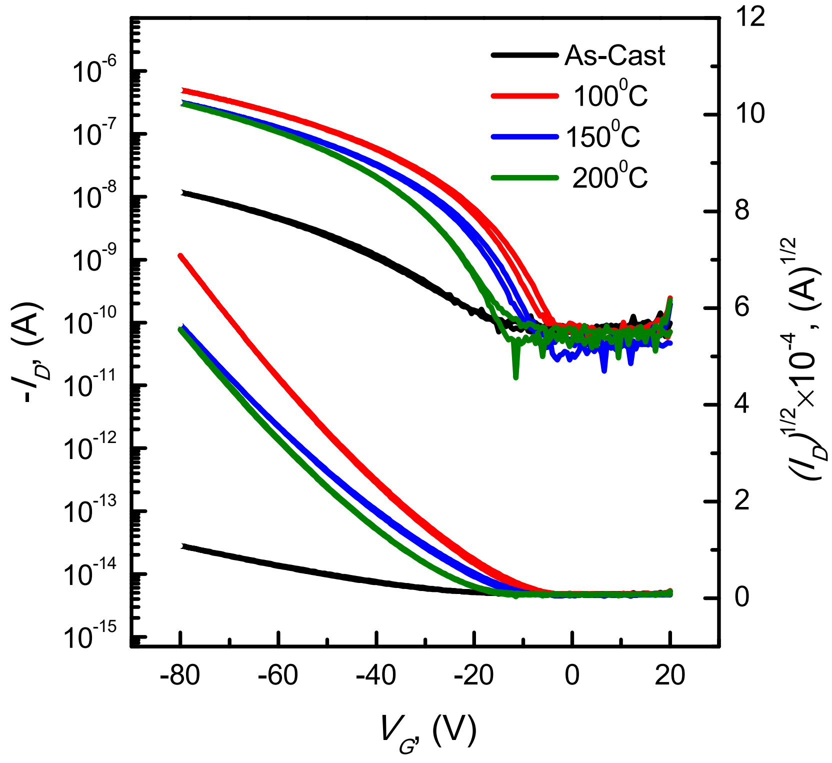

Figure S3. Transfer output characteristics of PTPABDT based OFETs with different annealing temperatures during device fabrication.

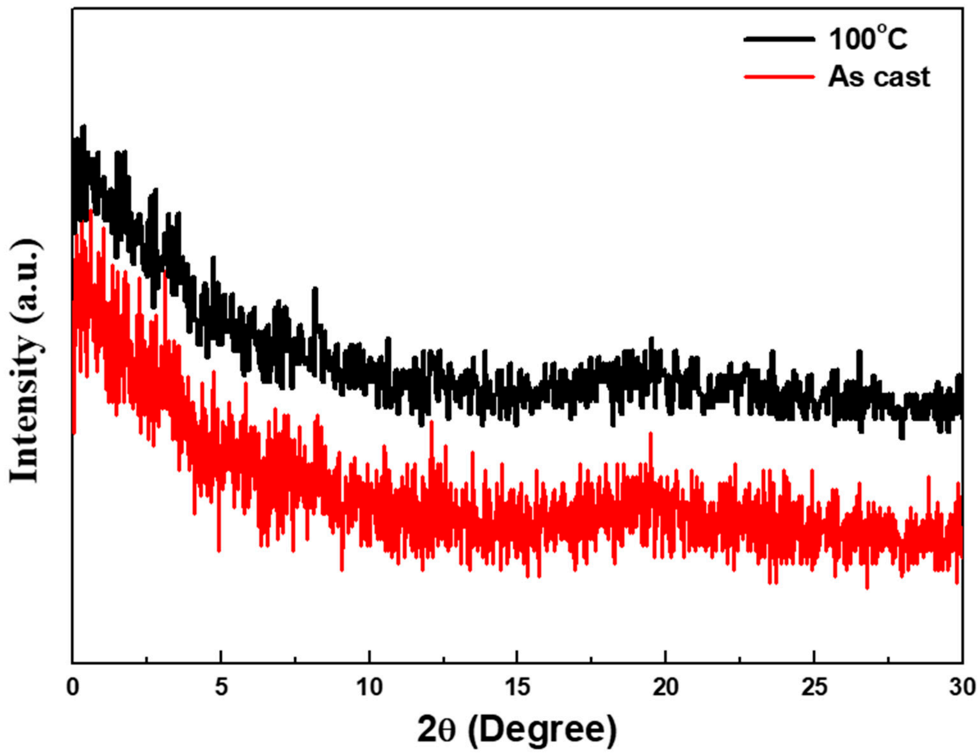

Figure S4. X-ray diffraction pattern at various annealing temperatures.
